# Recent Advances in Ultra-Weak Fiber Bragg Gratings Array for High-Performance Distributed Acoustic Sensing (Invited)

**DOI:** 10.3390/s26020742

**Published:** 2026-01-22

**Authors:** Yihang Wang, Baijie Xu, Guanfeng Chen, Guixin Yin, Xizhen Xu, Zhiwei Lin, Cailing Fu, Yiping Wang, Jun He

**Affiliations:** 1 State Key Laboratory of Radio Frequency Heterogeneous Integration, Key Laboratory of Optoelectronic Devices and Systems of Ministry of Education/Guangdong Province, College of Physics and Optoelectronic Engineering, Shenzhen University, Shenzhen 518060, China; 2 Shenzhen Key Laboratory of Photonic Devices and Sensing Systems for Internet of Things, Guangdong and Hong Kong Joint Research Centre for Optical Fibre Sensors, Shenzhen University, Shenzhen 518060, China

**Keywords:** distributed fiber sensor, ultra-weak fiber Bragg grating, distributed acoustic sensing

## Abstract

Distributed acoustic sensing (DAS) systems have been widely employed in oil and gas resource exploration, pipeline monitoring, traffic and transportation, structural health monitoring, hydrophone usage, and perimeter security due to their ability to perform large-scale distributed acoustic measurements. Conventional DAS relies on Rayleigh backscattering (RBS) from standard single-mode fibers (SMFs), which inherently limits the signal-to-noise ratio (SNR) and sensing robustness. Ultra-weak fiber Bragg grating (UWFBG) arrays can significantly enhance backscattering intensity and thereby improve DAS performance. This review provides a comprehensive overview of recent advances in UWFBG arrays for high-performance DAS. We introduce major inscription techniques for UWFBG arrays, including the drawing tower grating method, ultraviolet (UV) exposure through UV-transparent coating fiber technologies, and femtosecond laser direct writing methods. Furthermore, we summarize the applications of UWFBG arrays in DAS systems for the enhancement of RBS intensity, suppression of fading, improvement of frequency response, and phase noise compensation. Finally, the prospects of UWFBG-enhanced DAS technologies are discussed.

## 1. Introduction

Distributed optical fiber sensing (DOFS) employs optical fibers both as sensing elements and transmission media for external disturbances, enabling high-precision measurements of various environmental parameters [1,2]. As an important DOFS technology, distributed acoustic sensing (DAS), which utilizes the Rayleigh backscattering (RBS) signal for sensing, has undergone rapid development in recent years. Unlike traditional point-based acoustic sensors, DAS enables the long-range (tens of kilometers), high-density, real-time detection and localization of vibrational and acoustic events along an optical fiber by analyzing the phase and intensity of Rayleigh backscattering. This unique operating principle provides DAS with several unparalleled advantages. First, its fully distributed sensing capability eliminates blind spots, achieving true continuous coverage [3]. Second, the sensing fiber is passive, requiring no electrical power, and exhibits strong resistance to electromagnetic interference and corrosion, making it highly suitable for long-term, stable operation in harsh environments such as those involving flammable, explosive, or strong electromagnetic field conditions [4]. Furthermore, a single fiber-optic cable can replace thousands of conventional sensors, significantly reducing deployment costs and maintenance complexity for large-scale infrastructure monitoring. Leveraging these outstanding features, DAS technology is driving the intelligent transformation of numerous industries with unprecedented scope and depth. For example, in oil and gas applications, it is used for dynamic reservoir analysis and pipeline safety intrusion monitoring [5]. For structural health monitoring, DAS commonly detects inherent defects in structures and enables hazard warnings [6]. In the transportation area, it monitors railway track integrity and highway vehicle status to ensure operational safety [7,8].

However, DAS systems based on standard single-mode fibers (SMFs) exhibit several inherent limitations. The Rayleigh backscattering coefficient of SMF is relatively low, approximately −70 dB/m [9], resulting in a weak backscattering intensity and, consequently, a reduced signal-to-noise ratio (SNR). As optical power continuously attenuates along the fiber during propagation, the sensing range is constrained by detector sensitivity, and its upper limit is reached once the received signal power drops below the detector noise floor. This low SNR limits the sensing range and degrades detection sensitivity. Moreover, Rayleigh backscattering centers are densely distributed within SMFs, while the probe pulse exhibits a finite coherence length. Within this coherence length, the coherent superposition of RBS signals from discrete scattering centers can cause the received intensity at certain positions to approach zero, a phenomenon known as coherent fading, which leads to sensing dead zones [3]. Additionally, to distinguish the RBS generated by adjacent probe pulses, the time interval between probe pulses (i.e., acoustic wave sampling interval) must exceed the round-trip propagation time of light in the fiber, resulting in a fundamental trade-off between the sensing range and the acoustic response bandwidth of the system.

To overcome these limitations, scattering-enhanced arrays based on ultra-weak fiber Bragg gratings (UWFBGs) or scattering-enhanced points (SEPs) have been incorporated into optical fibers, significantly improving DAS performance. For example, the UWFBG array can provide much stronger backscattering signals than intrinsic Rayleigh scattering centers, thereby improving the SNR of the detected backscattering signal. This enhancement enables a longer sensing range and improves detection sensitivity [9,10]. Furthermore, the spacing between adjacent UWFBGs is typically set to exceed the coherence length of the probe pulse. This large spacing eliminates interference between the scattered light of adjacent fiber Bragg gratings (FBGs) and effectively suppresses coherent fading [11]. Moreover, this configuration introduces temporal gaps between the received backscattering signals of UWFBGs, which facilitates the implementation of time-slot multiplexing schemes to expand the system’s frequency response bandwidth [12]. In addition, benefiting from the sparse spatial distribution of the UWFBG array, the backscattering signal from each individual UWFBG can be extracted separately and employed as a dedicated reference structure to compensate for phase noise [13].

This paper reviews the performance enhancement mechanisms of Rayleigh scattering-enhanced arrays in DAS systems. The fabrication techniques for large-scale grating arrays, such as the drawing tower grating method, ultraviolet (UV) exposure through UV-transparent coating fiber technologies, and femtosecond (fs) laser direct writing methods, are introduced. We elaborate on the recent progress of UWFBG arrays in improving DAS performance. The UWFBG array improved optical SNR, suppressed fading, enlarged frequency response, and compensated phase noise. Furthermore, we demonstrate the representative applications of DAS based on scattering-enhanced arrays in the fields of oil and gas resource exploration, pipeline monitoring, traffic and transportation, structural health monitoring, hydrophones, and perimeter security and intrusion incidents. Finally, the prospects of UWFBG-enhanced DAS technologies are discussed.

## 2. Basic Principles

### 2.1. Principle of Conventional SMF-Based DAS

DAS technology can measure external dynamic strain by demodulating the phase change or frequency drift of the RBS signal. Acoustic or vibrational perturbations applied to the optical fiber produce dynamic strain, which changes both the refractive index and the effective optical path length through a photoelastic effect. These changes lead to the phase modulation of the RBS and can be expressed as [14,15,16]

(1) $$\Delta \varphi =\frac{4\pi nLv_{0}}{c}\left(1-p_{e}\right)\cdot \Delta \varepsilon ,$$

(2) $$\frac{\Delta v}{v_{0}}=-\left(1-p_{e}\right)\Delta \varepsilon \approx 0.78\cdot \Delta \varepsilon ,$$

where *v*_0_ is the center frequency of the laser and *p_e_* is an effective strain-optic constant defined as

(3) $$p_{e}=\frac{n^{2}}{2}\left[p_{12}-\xi \left(p_{11}+p_{12}\right)\right],$$

where *n* is the effective refractive index, *p*_11_ and *p*_12_ are components of the strain-optic tensor, and *ξ* is Poisson’s ratio. Once the phase difference between adjacent sensing points is extracted, the corresponding axial strain variation can be determined, enabling the quantitative reconstruction of acoustic or vibrational signals.

**Figure 1:**
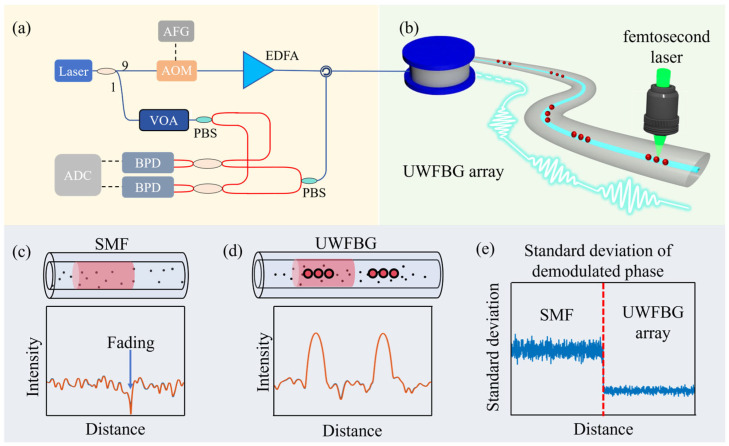
Schematic of sensing mechanism of DAS based on UWFBG array. (**a**) Experimental setup for COTDR system. (**b**) The fabrication of UWFBG array. Scattering models of (**c**) SMF and (**d**) UWFBG array. (**e**) The comparison of demodulated phase standard deviation between SMF and UWFBG array.

The DAS system interrogates the fiber under test (FUT) by emitting laser pulses. A standard coherent optical time-domain reflectometer (COTDR) system is shown in Figure 1a. The narrow-linewidth continuous-wave laser is modulated into a train of optical pulses by an acousto-optic modulator (AOM) and then launched into the FUT. As the laser pulse propagates through the fiber, RBS signals are continuously generated. Then, the RBS signals interfere with the local oscillator (LO) and are divided into two polarization states by polarizing beam splitters (PBS). The beat signals reach the photodetector with time delays corresponding to their respective scattering positions, thereby enabling the localization of vibrations along the fiber. The beat signal can be expressed as

(4) $$E(t)=\sum_{i=1}^{M}A_{i}W(\frac{t-\tau_{i}}{T_{p}})\cdot \exp\left\{j\left[2\pi f_{0}(t-\tau_{i})-\omega_{c}\tau_{i}+\Delta \varphi \right]\right\}\;,$$

where *W*(*t*) represents the window function, *M* represents the total number of scattering points, *T_p_* represents the pulse width, *A_i_* and *τ_i_* represent the amplitude and the round-trip delay of the *i*th scattering center, respectively, *f*_0_ represents the frequency shift caused by the modulator, *ω_c_* represents the center frequency of the laser, and Δ*φ* represents the phase change caused by strain. Through coherent demodulation schemes such as the COTDR [17], phase-generated carrier (PGC) [18], 3 × 3 coupler [19], and double pulse scheme [20], the phase can be demodulated and the acoustic waveform can be recovered.

It can be seen from Equation (4) that the RBS signal at each time is formed by the coherent superposition of scattering lights within a spatial range of *cT_p_*/2*n*. When these scattering light components interfere destructively, the RBS intensity at that location drops below the background noise level, leading to a loss of sensing capability. This phenomenon is known as coherent fading, as shown in Figure 1c. In addition, some algorithms, such as the inverse tangent algorithm and IQ demodulation algorithm, are commonly used to demodulate the phase. The noise of the demodulated phase is affected by the optical SNR, which can be expressed as [21]

(5) $$\sigma_{\varphi -a}\left(z\right)=\sqrt{\frac{1}{{\mathrm{SNR}}_{a}\left(z\right)}}=\sqrt{\frac{\sigma_{n}^{2}}{\langle A^{2}\left(z\right)\rangle }},$$

where *σ_φ−a_* denotes the phase precision limit imposed by additive noise, *SNR_a_*(*z*) and *A^2^*(*z*) represent the SNR and the optical signal power at position z, respectively, and *σ^2^_n_* is the complex noise variance dominated by thermal noise and shot noise. For SMF, the RBS signal is inherently weak, resulting in significant additive noise over a long sensing range, whereas UWFBG or SEP arrays exhibit high and stable reflections, effectively avoiding this issue.

### 2.2. Performance Enhancement Mechanisms of UWFBG in DAS

The reflectivity of an UWFBG is typically several orders of magnitude higher than the RBS coefficient of an SMF. Furthermore, the predesigned spacing between UWFBGs is larger than the average spacing of random Rayleigh scattering centers in SMF, as illustrated in Figure 1d. Hence, the reflection signals of UWFBGs are distinguishable in the time domain. For a quasi-distributed acoustic sensing (QDAS) system based on UWFBG arrays, the backscattering signal can still be represented using Equation (4). However, a key distinction lies in the origin of the signal: the target signal is generated by the UWFBGs, whereas the inherent RBS from the fiber is treated as a noise source [22]. If the spatial width of the optical pulse (or the effective pulse width after compression) is smaller than the grating spacing of the UWFBG array, the backscattering signals from individual UWFBGs do not temporally overlap, thus preventing coherent superposition and effectively mitigating coherent fading. Moreover, owing to the high reflectivity of UWFBGs, the energy of the backscattering signal is significantly enhanced, which effectively improves the SNR of the system. This enhancement suppresses the relative impact of additive noise and thereby extends the maximum sensing range, as shown in Figure 1e.

In addition, the large and deterministic spacing between UWFBGs allows each grating to be effectively resolved and individually addressed. This property enables advanced applications, such as in channel multiplexing technology and the phase noise compensation method. Channel multiplexing technology, including time-slot multiplexing, exploit the free space between UWFBGs by optimizing the pulse emission timing, thereby improving the frequency response bandwidth [12]. Moreover, a UWFBG array can serve as a reference structure to extract and compensate for the phase noise of the laser source [13].

## 3. Fabrication Methods of UWFBG Array

Conventional FBGs are prepared using a UV exposure method. However, the weak intrinsic photosensitivity of standard SMF requires the use of photosensitivity enhancement techniques such as hydrogen loading or boron doping [23,24]. These processes necessitate the removal of the fiber coating, which significantly reduces fabrication efficiency. Moreover, even after recoating, the mechanical strength of the fiber is still degraded. To address these issues, some methods have been extensively researched, including drawing tower grating (DTG) technology [25], the UV exposure method through UV-transparent coating fiber [26], and fs laser direct writing technology [27].

### 3.1. Drawing Tower Grating Method

**Figure 2:**
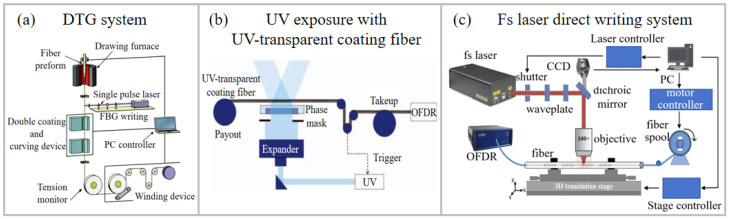
Schematic diagram of (**a**) drawing tower grating method [25], (**b**) UV exposure with UV-transparent coating fiber [26], and (**c**) fs laser direct writing technology [27]. Reproduced with permission from Gao et al. [25]. Copyright 2021 Springer Nature. Reproduced with permission from Westbrook et al. [26]. Copyright 2017 Institute of Electrical and Electronics Engineers Inc. Reproduced with permission from Xu et al. [27]. Copyright 2021 Optical Society of America.

As shown in Figure 2a, during the optical fiber drawing process, DTG technology fabricates gratings prior to the application of the fiber coating, thereby effectively avoiding the UV light absorption problem associated with the coating layer. DTG technology was first demonstrated by the University of Southampton in 1993 [28]. A KrF laser combined with a Talbot interferometer was used for in situ fabrication of FBGs during the fiber drawing process from preform rods prior to coating. This method ensures the mechanical strength of FBG and prevents the coating from absorbing the UV laser. However, this method demands an extremely stable optical path and is highly vulnerable to environmental disturbances, which degrade the quality of the fabricated FBGs. The DTG technology based on phase masks was proposed to overcome this problem [29,30]. It utilizes the interference field generated directly behind the phase mask to inscribe FBG arrays. The fabricated large-scale grating array exhibited excellent wavelength consistency (with a deviation of <0.1 nm). However, the fabricated FBG array exhibited poor reflection uniformity. In 2015, researchers theoretically analyzed the underlying cause of spectral distortion in UWFBGs [31]. It was identified that the primary issue resulted from poor laser spot quality, specifically the random non-uniformity in its intensity profile, which introduced inconsistent refractive index modulation. By adjusting the laser beam parameters to achieve a highly uniform intensity profile, the researchers successfully fabricated a high-performance FBG array with significantly improved spectral characteristics.

To increase the integration number of UWFBGs, suppressing significant crosstalk induced by spectral shadowing and multiple reflections is essential [32]. A major solution was demonstrated in 2017, where phase mask technology enabled the fabrication of a dense array of 6680 UWFBGs with a center-to-center spacing of 1.5 mm over a 10 m fiber [33]. In this work, crosstalk from spectral shadowing and multiple reflections was effectively mitigated by carefully optimizing the center wavelengths and spacings of the UWFBGs. Subsequently, a more advanced approach employed UWFBG arrays with randomized center wavelengths and grating spacings, further reducing crosstalk [34]. A breakthrough was achieved in 2022 with the successful fabrication of an array consisting of over 10,000 multi-wavelength UWFBGs [25]. This array exhibited a uniform reflectivity of −40 dB and an 8.14 dB power difference between the intended UWFBG signals and ghost gratings, thereby effectively suppressing crosstalk.

Furthermore, in order to increase the high-temperature sensing performance of DTG, researchers at Wuhan University of Technology fabricated an FBG array in Polyimide (PI)-coated fibers with hydrogen loading. After high-temperature annealing, the fabricated FBG array could work at a temperature of 350 °C [35,36]. Moreover, additional hydrogen loading and thermal regeneration processes were used to achieve a thermally regenerated DTG, which can withstand high temperatures up to 900 °C. However, these thermally regenerated DTGs always exhibited higher reflectivity, which is not conducive to the large-scale integration of gratings [37].

### 3.2. UV Exposure with UV-Transparent Coating Fiber

Another major approach to fabricating grating arrays is UV exposure with UV-transparent coating fibers. This method eliminates the UV absorption present in conventional fiber coatings, as shown in Figure 2b. In 2014, OFS Laboratories in the United States employed roll-to-roll technology combined with phase mask processing to fabricate a continuous ultra-weak grating array on twisted multi-core optical fibers coated with UV-transparent material. The fabricated FBGs exhibited a reflectivity approximately 25 dB higher than that of the RBS [38]. However, the total fiber length was only about 2 m. In 2017, the same group used a chirped phase mask to simultaneously fabricate continuous grating arrays on all cores of twisted multi-core fibers [39]. The reflection was approximately 20 dB higher than that of the RBS over a bandwidth of 25 nm. Moreover, they subsequently fabricated a 1 km long continuous weakly chirped grating array on an SMF coated with UV-transparent material [26]. The scattering intensity was approximately 14 dB higher than that of the RBS, with a bandwidth of 10 nm. In 2021, a high-performance fiber coating with a UV transmittance of 80% was developed by the Huazhong University of Science and Technology [40]. The UWFBG array with 5 m spacing and 10 mm spacing was successfully fabricated, meeting the requirements of different spatial resolution applications [41]. Moreover, a full-link SNR equalization system based on a gradient discrete scattering enhanced fiber was proposed. The backscattering rate gradually increases the as optical loss grows with the fiber length, thereby dynamically balancing optical attenuation with the scattering enhancement effect [42]. Moreover, they achieved the inscription of a UWFBG array with mixed position–wavelength encoding [43].

However, UV-laser-induced Type-I FBGs are prone to being erased at temperatures above 450 °C [1]. This limitation restricts their applicability in extreme environments such as hypersonic aircraft, aircraft engines, nuclear reactors, power plants, and metallurgical furnaces [44,45].

### 3.3. Femtosecond Laser Direct Writing Method

Fs lasers, featuring an ultra-short pulse width and extremely high peak intensity, are powerful tools for fabricating various FBG arrays. Compared to UV exposure technology, the fs laser direct writing method does not rely on the photosensitivity of fibers and can fabricate high-quality grating on almost all types of materials [46]. Moreover, Type-II FBGs with permanent structural changes can be inscribed using high-intensity fs lasers and can withstand a high temperature of above 1000 °C.

As shown in Figure 2c, the coated fiber is fixed on a precision displacement platform, and the fs laser is focused on the fiber core. Combined with the reel-to-reel system, a large-scale grating array can be achieved. We have developed an automated fs laser system for fabricating large-scale UWFBG arrays. The system consists of three key components: an fs laser microfabrication setup, a fiber core recognition system, and a fiber-optic reel-to-reel system. The fabrication process of the UWFBG arrays is as follows: first, the fiber core is identified by analyzing the core/cladding boundaries in top-view microscope images [47]. This is achieved through a Gaussian smoothing algorithm followed by a grayscale intensity profile extraction. Additionally, the grayscale of fiber is scanned along the cross section, and an autocorrelation algorithm of the grayscale intensity is performed to locate the autocorrelation peak, which corresponds to the fiber core’s center along the cross section. Subsequently, the high-precision 3D air floating translation stage aligns the center of the fiber core with the laser beam. The automated preparation of UWFBG arrays is further facilitated by the fiber reel-to-reel system, and the large-scale gratings could be integrated.

Using the aforementioned system, we successfully induced permanent SEPs in an SMF with a spacing of 233 µm. The SEPs exhibited a 26 dB enhancement in RBS and a 0.6 dB insertion loss [48]. Moreover, a permanent SEP array was inscribed using an fs laser in each slightly twisted core of a multi-core fiber (MCF) [49], with an interval of 200 µm. Two SEPs can form an in-fiber microcavity (MC), featured by an intrinsic Fabry–Perot interferometer (IFPI). We successfully fabricated a large-scale multiplexed high-density weak-fiber MC array with a cavity length of only 100 µm and a peak reflectivity as low as −55 dB [50]. In addition, by controlling the cavity length of the MC, the reflectivity and free spectral range (FSR) can be adjusted, and the insertion loss of such devices can be as low as 0.0009 dB [51]. Furthermore, composite microcavity arrays (CMCA) with a narrower bandwidth and a spatial spacing of 1 mm were inscribed, effectively improving sensing accuracy. Periodic SEPs can form a grating. We successfully fabricated 200 identical 120th-order FBGs with a spacing of 10 mm on a 2 m long SMF via the fs laser point-by-point (PbP) writing technology [27]. The peak reflectivity was approximately −45 dB. Experimental results indicate that the thermal repeatability of UWFBGs is superior at a high temperature of 1000 °C. Notably, we fabricated an ultra-short UWFBG array with a length of 30 μm and a bandwidth of 24.6 nm, enabling its application in a wide temperature range, since the wavelength mismatch between the grating and the laser source can be avoided [52]. After optimizing the fabrication parameters, such as fs laser energy, grating length, grating order, and offset distance, the large-scale UWFBG array with an amount of 10,000 was achieved.

Other research groups are also making efforts in the automated fabrication of gratings. A sleeve-based automatic alignment system was introduced to compensate for the position fluctuations of the fiber core relative to the fs laser [53]. The high-quality FBGs with lengths from 0.1 to 50 mm were fabricated. Moreover, image recognition technology was used to achieve precise spatial automatic alignment [54]. A uniform FBG with a length of 50 mm was successfully fabricated on a commercial high-numerical-aperture (NA) fiber. The accuracy is only limited by diffraction.

The U.S. Naval Research Laboratory used an fs laser to fabricate an SEP array on the SMF cladding, with a reflectivity of −53 dB and a loss of only 0.0001 dB in 2020 [9]. Moreover, Northwest University studied the insertion loss and reflection spectra of off-axis FBGs. The insertion loss and reflection intensity gradually decreased with increasing offset. The insertion loss can be reduced to 0.00001 dB [55,56]. In addition, Shanghai Jiao Tong University optimized the scattering structure as a plane composed of multiple refractive index modulation lines to reduce the insertion loss [57]. They achieved a transmission loss of 0.34 dB/km with a length of 9.8 km, a spacing of 10 m, and a reflectivity of about −42 dB.

The technical characteristics, advantages, and typical applicable scenarios of these three fabrication technologies are summarized in Table 1. DTG technology employs in situ UV phase mask inscription or UV Talbot interferometer inscription during the fiber drawing process prior to coating. This technology preserves the mechanical strength of the fiber, avoids UV absorption with its coating, and exhibits a highly efficient fabrication. This makes it particularly suitable for distributed fiber sensing in large-scale and long-distance systems. Alternatively, UV exposure through UV-transparent coating enables grating inscription without the need for coating removal, offering a mature and stable fabrication method for distributed sensing in conventional industrial environments. In contrast, fs laser direct writing technology, utilizing point-by-point or plane-by-plane inscription through standard fiber coating, does not rely on fiber photosensitivity and provides high design flexibility along with enhanced high-temperature resistance. This technique is especially advantageous for distributed fiber sensing in extreme environments.

## 4. High-Performance DAS Using UWFBG

### 4.1. Enhancement of Sensing Range and Sensitivity

**Figure 3:**
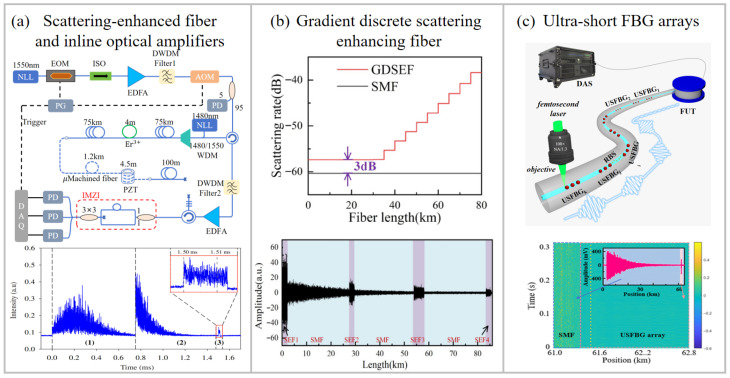
Schematic diagram of representative techniques for extending the sensing range in DAS systems: (**a**) micro-machined scattering-enhanced fiber combined with inline amplification method [10], (1) RBS of the first 75 km SMF, (2) RBS of the fiber after inline optical amplification, (3) RBS of the micro-machined scattering-enhanced fiber, (**b**) gradient discrete scattering-enhanced fiber technology [41], and (**c**) ultra-short FBG arrays method [52]. NLL: narrow-linewidth laser; EOM: electro-optic modulator; EDFA: erbium-doped fiber amplifier; PG: pulse generator; PD: photodetector; PZT: piezoelectric transducer; IMZI: imbalanced Mach–Zehnder interferometer. Reproduced with permission from Masoudi et al. [10]. Copyright 2021 Optical Society of America. Reprinted with permission from Fan et al. [41]. Copyright 2023 for the corresponding author Hao Li. Reproduced with permission from Xu et al. [52]. Copyright 2025 Optical Society of America.

Compared to the RBS in standard SMFs, UWFBG arrays exhibit a significantly higher backscattering coefficient, which effectively enhances the SNR of the scattering light, thereby extending the sensing range and suppressing phase noise induced by low light intensity. In 2020, a QDAS system based on dual-chirp pulses and WFBGs achieved a sensing range of 101.64 km with a spatial resolution of 10 m [58]. Moreover, in 2021, by combining inline optical amplification with a micro-machined scattering-enhanced fiber, a DAS system with a detection range exceeding 150 km was realized, as shown in Figure 3a. This system achieved a strain resolution of 33 nε/√Hz at 1 Hz and a spatial resolution of 5 m [10]. However, the aforementioned approaches only employed scattering-enchanted fiber at the fiber’s distal end, with a limited number of scattering points. To mitigate this limitation, a high-capacity DAS system incorporating 10,828 UWFBGs was proposed in 2022 [59]. This system achieved a sensing range of 54.14 km, a strain sensitivity of 189.54 pε/√Hz, and a spatial resolution of 5 m. Furthermore, a full-link SNR-equalized DAS system was developed using gradient discrete scattering-enhanced fiber, as shown in Figure 3b [41]. In this system, the backscattering rate gradually increased along the fiber length, achieving full-link SNR-equalized acoustic detection with a range of approximately 80 km, a strain resolution of 15 pε/√Hz at 10 Hz, and a spatial resolution of 5 m.

In order to address the problem of severe additive noise in SMF, researchers have conducted a lot of work. A 300-element UWFBG array was integrated into a COTDR system, demonstrating a 9 dB enhancement in RBS intensity over SMF [60]. This approach was further validated in a dual-pulse DAS system, where the strain sensitivity was 0.15 pε/√Hz with a spatial resolution of 20 m. The noise was suppressed by approximately 20 dB compared to a conventional SMF-based system [9]. Subsequent research delved deeper into the noise-reduction mechanism. Analyses of the signal and noise components in both SMF-based and UWFBG-based phase-sensitivity optical time-domain reflectometer (Φ-OTDR) systems revealed that reflectors with higher peak reflectivity exhibit a lower noise floor [61,62]. Furthermore, studies established that the system noise level decreases exponentially with increasing grating reflectance [63,64]. For instance, when the reflectivity was raised from −63 dB to −48 dB, the sensitivity improved from 0.238 pε/√Hz to 0.095 pε/√Hz with a spatial resolution of 10 m. To further enhance the sensitivity of DAS systems, the UWFBG array was employed in conjunction with chirped-pulse compression techniques. These techniques effectively increased the SNR by integrating the energy of a long probe pulse upon reception. In 2019, a DAS system integrating chirped-pulse compression and phase noise compensation was demonstrated, utilizing a scattering-enhanced fiber with a reflectivity of −40 dB and a length of 20 km. By combining these techniques, a noise floor of 92.84 fε/√Hz was achieved with a spatial resolution of 20 m [65]. In addition, the integration of zero-insertion coding with UWFBG technology achieved a sensitivity of 58.3 pε/√Hz and a spatial resolution of 5 m over a 1.5 km sensing range [66].

We proposed and demonstrated a high-performance DAS system using ultra-short fiber Bragg grating arrays (USFBG) and an optical pulse compression algorithm, as shown in Figure 3c. A long-distance DAS with a sensing range of 60 km, a spatial resolution of 5.9 m, and a strain resolution of 13.9 pε/√Hz was achieved [52]. Moreover, a high spatial resolution DAS system was also demonstrated using an SEP array. This system exhibited a noise floor of 1.46 nε/√Hz with a spatial resolution of 1.2 cm [67]. Furthermore, the use of the UWFBG array mitigated Doppler crosstalk in the chirped-pulse Φ-OTDR system, resulting in a 20 dB increase in the crosstalk suppression ratio and a sensitivity of 190 pε/√Hz with a spatial resolution of 10 cm [68]. Combined with a seven-core FBG, a vectorial DAS was achieved with a spatial resolution of 1.1 m, a strain resolution of 1.1 pε/√Hz, and a measurement error of the acoustic azimuth angle of only 1.92° [69].

### 4.2. Suppression of Fading

In standard SMF, RBS signals from different scattering centers have a probability of coherent cancelation at certain positions, causing coherent fading. In addition, random polarization fluctuations lead to polarization fading. These effects seriously limit the sensing performance. In DAS systems employing UWFBG arrays, the discrete reflections from the gratings are separated during demodulation, thereby eliminating coherent fading, although polarization fading still remains.

**Figure 4:**
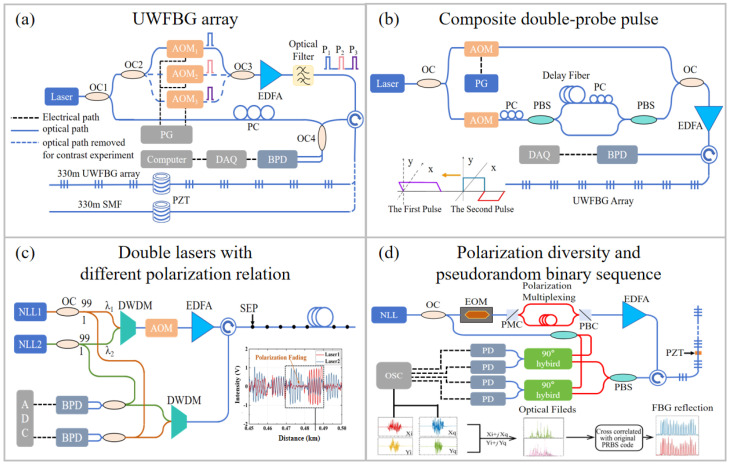
Schematic diagram of fading-suppression techniques in DAS systems: (**a**) UWFBG array [11], (**b**) CDPP technology [71], (**c**) dual-laser scheme employing orthogonal polarization states [72], and (**d**) polarization-diversity reception [74]. BPD: balanced photodetector. PC: polarization controller. OSC: oscilloscope. PMC: polarization maintaining coupler. PBC: polarization beam combiner. Reproduced with permission from Wang et al. [71]. Copyright 2019 Optical Society of America. Reproduced with permission from Liu et al. [72]. Copyright 2020 to the corresponding author Qizhen Sun. Reproduced with permission from Zhang et al. [74]. Copyright 2023 Optical Society of America.

Figure 4a shows the COTDR system using an SMF or UWFBG array. The use of a UWFBG array significantly reduced the fading probability in SMF-based DAS [11]. To mitigate polarization fading, polarization-maintaining fiber systems and polarization-diversity detection techniques have been developed [70]. In addition, composite double-probe pulse (CDPP) schemes also provide effective fading suppression. A long-duration pulse was combined with two short consecutive pulses possessing orthogonal polarization states, as shown in Figure 4b [71]. Figure 4c shows that polarization fading can also be eliminated by synthesizing UWFBG backscattering signals generated by two lasers with different polarization states, leading to a 19.62 dB SNR improvement in fading-affected channels [72]. Moreover, polarization-diversity receivers can retrieve the phase changes induced by local birefringence and compute the polarization-independent phase through vector operations, thereby effectively suppressing polarization fading [73]. Furthermore, two orthogonally polarized, phase-coded pulses separated by a fixed delay can be used to reconstruct the Jones-matrix components of the UWFBG array, from which the vibration-induced polarization-independent phase can be obtained, as shown in Figure 4d [74].

### 4.3. Improvement of Frequency Response

In DAS systems, the acoustic sampling interval is constrained to be longer than the round-trip propagation time of light in the optical fiber, thereby limiting the achievable frequency response range. To improve the frequency response range, UWFBG arrays can be combined with channel multiplexing technologies such as frequency division multiplexing (FDM), wavelength division multiplexing (WDM), time-slot multiplexing (TSM), and code division multiplexing (CDM) technology.

#### 4.3.1. UWFBG Array with FDM and WDM Technology

FDM technology is a traditional approach to improve the frequency response of DAS [75], and it has some unique applications in UWFBG-based DAS systems. For example, in the direct detection scheme based on WFBGs, FDM can be achieved by using multiple groups of heterogeneous frequency dual pulses with different beat frequencies [76]. Acoustic sensing with a bandwidth of 2 kHz was realized on a 70 km sensing fiber with a spatial resolution of 10 m. The response bandwidth was improved by four times. In order to further improve the spectrum utilization efficiency, orthogonal frequency division multiplexing (OFDM) technology was proposed to multiplex multiple probe pulses into the same interrogation period [77]. The response bandwidth was improved by four times. To achieve the orthogonality between each signal, the signal of each reflector should have a rectangular-pulse-shaped envelope. Therefore, the OFDM method can be only used in the reflector array-based DAS system.

**Figure 5:**
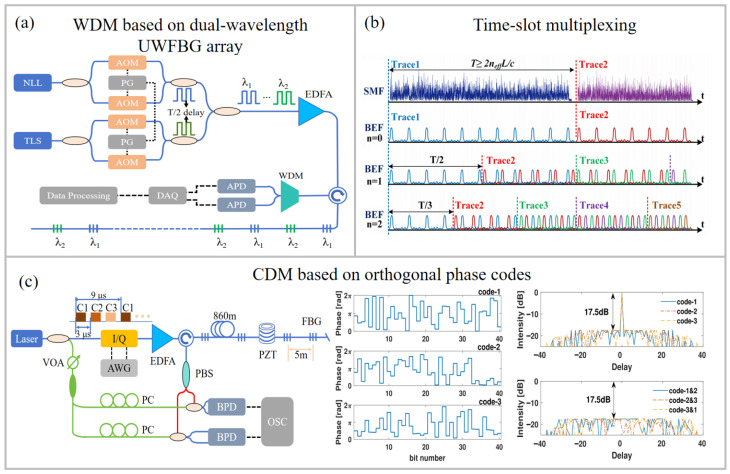
Schematic diagram of enhancing frequency response techniques: (**a**) WDM based on dual-wavelength UWFBG array [78], (**b**) time-slot multiplexing [12], and (**c**) CDM based on orthogonal codes [85]. I/Q: in-phase and quadrature modulator; AWG: arbitrary waveform generator; APD: avalanche photodiode. Reprinted with permission from Li et al. [12]. Copyright 2021 Springer Nature. Reproduced with permission from Jiang et al. [85]. Copyright 2021 Institute of Electrical and Electronics Engineers Inc.

In addition, WDM technology was also used to improve the frequency response. As shown in Figure 5a, a DAS system with an alternating dual-wavelength UWFBG array was proposed [78]. By launching dual-wavelength probe pulses with staggered emission times, the maximum sampling rate limited by the fiber length was doubled. Consequently, the spatial resolution improved from 10 m to 5 m, and both the dynamic strain range and the frequency-response range were doubled.

#### 4.3.2. UWFBG Array with TSM Technology

A UWFBG could be used to extend the frequency response bandwidth by using its intrinsic sparse distribution along optical fiber. Specifically, the space between adjacent gratings, corresponding to their time slot, can be used to insert and multiplex pulses, as shown in Figure 5b. The multiplexing capacity was constrained by the ratio between the FBG spacing and the main lobe width of the probe pulse. In 2020, interleaved chirped pulses (ICP) were used to interrogate UWFBG arrays, yielding a threefold enhancement in the frequency-response bandwidth [79]. Additionally, since the multiplexed probe pulses of the ICP method are in different frequency bands, they require extra frequency resources, which prevents the effective improvement of spatial resolution. To mitigate this problem, interleaved identical chirped pulses (IICP) using the same frequency band were employed, achieving a fivefold enhancement in the frequency-response bandwidth, a spatial resolution of 5 m, and a strain sensitivity of 2.8 pε/√Hz [80].

For further simplification, a TSM scheme using single-frequency pulses was proposed [12]. This method achieved a sixfold bandwidth enhancement using an SEP array with an interval of 200 ns and a pulse width of 30 ns. In addition, the coded array matched interrogation (C-AMI) integrated pulse-coding techniques with the optimized interpolation of SEPs. This method improved the SNR and achieved an interrogation rate of 5 MHz, which is 200 times higher than the conventional limit. It has also been applied to underwater ultrasonic sensing [81,82]. To address inconsistent phase shifts in multi-frequency multiplexing, a multi-frequency time-slot multiplexing (MFTSM) scheme was introduced [83]. The response bandwidth is tripled. It employed odd-numbered single-frequency pulses and even-numbered dual-frequency pulses to compensate for phase errors, while simultaneously alleviating the difficulty of locating UWFBGs in time-slot-based interrogation.

#### 4.3.3. UWFBG Array with CDM Technology

As illustrated in Figure 5c, the CDM scheme employs orthogonal codes for multiplexing. Compared with the aforementioned methods, CDMs operating in the same frequency band prevent the degradation of spatial resolution. However, the sidelobes of the autocorrelation and cross-correlation functions will overlap with the main lobe, leading to undesired crosstalk. Owing to the inherently low spatial duty cycle, the UWFBG array could effectively reduce sidelobe overlap, thereby suppressing crosstalk [84,85]. Furthermore, empirical mode decomposition (EMD) was employed in the CDM system to remove low-frequency noise from each channel [86]. A response range of 1 Hz to 10 kHz was demonstrated at the far end of a 99.4 km fiber, extending the response bandwidth by a factor of 20. The achieved sensitivity is 33 pε/√Hz with a spatial resolution of 10 m. Furthermore, researchers from the University of Electronic Science and Technology of China employed a mismatch filtering scheme for orthogonal CDM technology, thereby suppressing crosstalk and improving the frequency response by three times in a standard SMF [87]. Moreover, by combining FDM, CDM, and chirped-pulse compression techniques, we achieved a spatial resolution of 10 cm for ultrasonic detection on a 30 m UWFBG array at the end of a 900 m SMF.

### 4.4. Phase Noise Compensation

Laser phase noise and frequency drift can also degrade the SNR of DAS systems besides the limitation imposed by low optical power [88]. Effective laser noise compensation is essential and can be implemented by using reference structure methods, optimized direct detection schemes, or phase noise compensation (PNC) digital algorithms.

#### 4.4.1. Phase Noise Compensation Based on Auxiliary Structures

**Figure 6:**
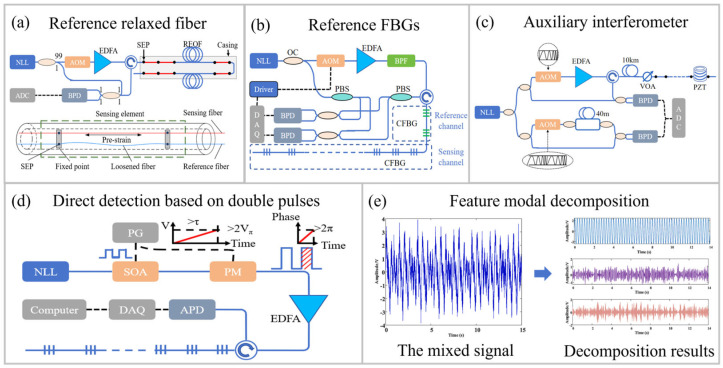
Schematic diagram of PNC methods based on auxiliary structure methods: (**a**) reference relaxed fiber [91], (**b**) reference chirped FBGs [13], and (**c**) auxiliary interferometer [65]. (**d**) Direct detection method based on double pulses [98]. (**e**) Feature modal decomposition method [100]. SOA: semiconductor optical amplifier; PM: phase modulator. Reproduced with permission from Liu et al. [91]. Copyright 2021 Institute of Optics and Electronics, Chinese Academy of Sciences. Reproduced with permission from Wu et al. [65]. Copyright 2019 Optical Society of America. Reprinted with permission from Wang et al. [100]. Copyright 2025 Elsevier B.V.

In a COTDR system, the LO interferes with the RBS signal. This interference enhances the detected signal intensity but simultaneously introduces laser phase noise [88]. By employing auxiliary structures to monitor the phase, this noise can be compensated. Weak reflection points were used in DAS systems to suppress phase noise over a sensing range of 30 km with a spatial resolution of 10 m [89]. Similarly, a reference FBG was used in single-shot chirped-pulse Φ-OTDR to track the laser frequency drift, improving low-frequency sensitivity and enabling the reconstruction of 1 Hz and 0.01 Hz dynamic strains with a standard deviation of 66 nε [90]. To further enhance compensation accuracy, a strain-free SEP array was adopted as a reference, as shown in Figure 6a. In addition, a least squares support vector machine (LS-SVM) was used to reduce residual errors arising from thermal-hysteresis mismatch between the sensing and reference fibers [91]. This method achieved a sensitivity of 10.5 pε/√Hz above 10 Hz and obtained a strain resolution of 166 pε at 0.001 Hz with a spatial resolution of 5 m. However, the above reference structures often require multiple reflection points or must be placed at the end of the FUT, limiting practicality. To improve applicability, a reference channel composed of two chirped FBGs was positioned at the front end of the FUT, as shown in Figure 6b [13]. Combined with a normalized least-mean-square adaptive filtering algorithm, this setup effectively compensated laser phase noise, achieving sensitivities of 34.5 pε/√Hz from 1 mHz to 1 Hz and 1.9 pε/√Hz from 1 Hz to 5 kHz with a spatial resolution of 5 m. To further compensate phase noise, an auxiliary interferometer with a UWFBG array was employed to monitor the LO phase, as shown in Figure 6c. A low-noise DAS system exhibited noise levels of 3.84 pε/√Hz at 10 Hz and 92.84 fε/√Hz over a frequency range of 500–2500 Hz while maintaining a spatial resolution of 20 m [65,92].

#### 4.4.2. Common-Mode Noise Elimination by Direct Detection

Compared with COTDR, direct detection methods do not require an LO, thereby reducing the influence of laser phase noise. Moreover, in conventional interferometer-based direct detection systems, two RBS signals generated from the same pulse or two pulses with short temporal delay interfere. As a result, the common-mode noise of a probe pulse can also be eliminated [93,94]. In these systems, the optical path difference (OPD) between the two interferometer arms should match the OPD between adjacent UWFBGs [95]. This constraint was removed by introducing adaptive-delay interferometry, in which the delay between dual-probe pulses was tuned dynamically in dual-pulse DAS. This technique achieved an SNR improvement exceeding 8 dB [96]. Furthermore, a three-step phase-shifted double-pulse method was proposed [97]. Three probe pulses with mutual phase shifts of 2π/3 enabled phase demodulation without a 3 × 3 coupler interferometer, yielding a noise level of 76.89 dB re rad^2^/Hz with a spatial resolution of 50 m. In addition, a linear-phase-modulated double-pulse scheme combined with an N-step phase-shift demodulation algorithm was introduced, as shown in Figure 6d [98]. This method significantly suppressed low-frequency self-noise, achieving an overall noise level of −54.3 dB re rad^2^/Hz with a spatial resolution of 10 m.

#### 4.4.3. Noise Component Decomposition and Compensation

PNC schemes based on feature modal decomposition (FMD) have also been developed. A combination of the RIME optimization algorithm and successive variational mode decomposition (RIME-SVMD) was introduced to suppress noise introduced by the light source and electronic components during 3 × 3 demodulation [99]. In this method, the intrinsic mode function (IMF) with the highest correlation coefficient was retained for signal reconstruction. The system achieved a 10 dB reduction in noise without compromising acoustic information. Furthermore, an FMD-based self-referencing scheme was employed to separate noise and signal components, effectively enhancing the low-frequency SNR, as illustrated in Figure 6e [100]. A reference interferometer was used to characterize system noise, and the FMD parameters were adaptively configured to preserve vibration components. This approach achieved an 18.1 dB improvement in sensitivity and an SNR of 70.1 dB at 1 Hz. Furthermore, variational mode decomposition (VMD) was incorporated into an auxiliary interferometer-based system to enable full-channel PNC [101]. A vibration sensitivity of 0.8 pε/√Hz was achieved with a spatial resolution of 20 m over a range of 500–2500 Hz by using an 1100 m UWFBG array. The overall SNR was improved by more than 20 dB compared with conventional Φ-OTDR using single-frequency pulses.

## 5. Applications of UWFBG-Based DAS

### 5.1. Oil and Gas Resource Exploration

Compared with conventional vertical seismic profiling (VSP) using downhole three-component (3C) geophone arrays, DAS enables seismic-wave detection using only a single standard SMF, offering low-cost, high-efficiency, and dense spatial sampling.

**Figure 7:**
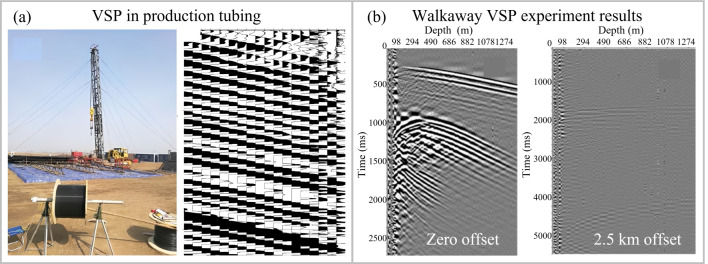
Application of UWFBG-based DAS in oil and gas resource exploration. (**a**) VSP in production tubing application [102]. (**b**) A walkaway VSP application [103]. Reproduced with permission from Li et al. [102]. Copyright 2019 to the corresponding author Minghong Yang. Reproduced with permission from Li et al. [103]. Copyright 2022 John Wiley and Sons.

UWFBG-based DAS systems have been applied to oil and gas exploration. In 2019, as shown in Figure 7a, a 1 km fiber-optic cable incorporating a UWFBG array fabricated via the DTG method, with 5 m sensor spacing, was deployed for seismic data acquisition. The system recorded seismic waves generated by controlled explosions. Figure 7a also presents the DAS-VSP results, where the sensor-dependent arrival times and high waveform consistency demonstrated system reliability [102]. In 2021, Sun et al. permanently installed a 1.4 km scattering-enhanced fiber-optic cable behind the casing in an oil field to monitor seismic waves induced by vibrators and explosive sources at various source–wellhead offsets. The DAS system acquired VSP data with a 2 m spatial sampling interval. Figure 7b shows that, when the source was near the wellhead, zero-offset VSP data exhibited a high SNR of 25 dB, and both upgoing and downgoing waves were clearly resolved. At a 2.5 km offset, the data revealed distinct direct P- and S-wave arrivals [103].

### 5.2. Pipeline Monitoring

**Figure 8:**
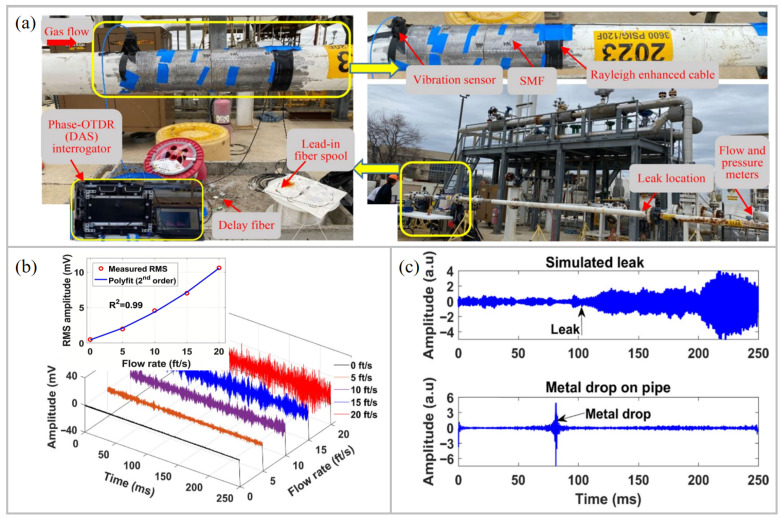
Application of UWFBG-based DAS in pipeline monitoring [106]. (**a**) Sensor deployment layout. (**b**) Flow rate measurement. (**c**) Leak and foreign object detection. Reproduced with permission from Lalam et al. [106]. Copyright 2023 Springer Nature.

Leak detection and the localization of oil and gas pipelines are key issues in pipeline condition monitoring. The long-distance acoustic detection characteristics of DAS technology have outstanding advantages in the field of pipeline monitoring. A DAS system based on a gradient-reflectance UWFBG array was employed to simulate pipeline leakage under high-pressure conditions in the laboratory [104]. The vibration signals induced by gas leaks were monitored, and leakage size was quantitatively estimated using the standard deviation (SD) of measured signals [105]. The SEP arrays were arranged in a spiral configuration to form a sensor array. A quadratic relationship between SD and leak size was observed; for a micro-leak of 0.8 mm, the error was only 0.03 mm. Leakage location was determined from the time delays of acoustic signals between channels using a fitting algorithm, achieving a positioning error of 3.85 cm. To meet field requirements for compressive and tensile strength, the scattering-enhanced fiber was embedded in a tight-buffered cable [106]. Figure 8a shows that the system was validated on a 4-inch steel gas pipeline under a fixed pressure of 1000 psi. The RMS of measured vibrations exhibited a quadratic relationship with flow rates ranging from 5 to 20 ft/s, as shown in Figure 8b. Moreover, leak and metal drop events were detected, as shown in Figure 8c.

To suppress external interference and extract leak signals accurately, a data-processing method combining spectral Complete Ensemble Empirical Mode Decomposition with Adaptive Noise (CEEMD) and wavelet transform was applied [107]. Mode decomposition and denoising effectively distinguished leak signals from interference, significantly reducing misjudgment. Furthermore, an improved convolutional neural network (CNN) incorporating a 2D squeeze-and-excitation (SE) attention mechanism was introduced for gas leak detection [108]. Experimental results showed that the optimized model achieved an average leak recognition accuracy of 95.33%. In addition, flow measurement and sand–water two-phase flow monitoring was achieved using DAS-based acoustic imaging, enabling 24 h flow profiles of natural gas pipelines with 98% accuracy and the quantitative detection of sand concentrations as low as 0.02 wt.% at 2.5 m/s [109,110].

### 5.3. Traffic and Transportation

Early detection of rail defects and structural damage is crucial for railway safety monitoring. Existing methods, however, cannot satisfy the requirements for real-time, high-precision detection. DAS can effectively record the instantaneous elastic waves generated by the interaction between the wheel and rail to monitor the wheel–rail relationship. The elastic waves propagate in both directions along the track. By detecting the time delay of waves in each channel, defects can be located. In particular, a dual-frequency joint-processing algorithm was proposed to improve location accuracy [111], achieving a standard deviation of 0.314 m in tests. In addition, artificial intelligence algorithms have advantages in pattern recognition [112]. A method based on the convolutional autoencoder (CAE) network was proposed for feature extraction and the recognition of loose fasteners [113]. Integrating the CAE with a pseudo-Hilbert scan algorithm lead to an improvement of 23.8% in identification accuracy. For train operation monitoring, a real-time streaming data-processing scheme combining singular value decomposition (SVD) and the sequential similarity detection algorithm (SSDA) was proposed [114]. Vibration signals were first denoised and converted into grayscale images. A template library for image matching was then constructed using SVD and texture features, improving recognition efficiency. Experiments with actual train data showed that the SVD-SSDA system using UWFBG arrays effectively meets real-time train tracking requirements. An abnormal wheel–track relationship recognition scheme was also proposed [115]. Vibration signals detected by sensors were decomposed using VMD. Abnormal signals are identified by thresholding, and the extreme points of abnormal intrinsic mode functions locate abnormal bogies.

**Figure 9:**
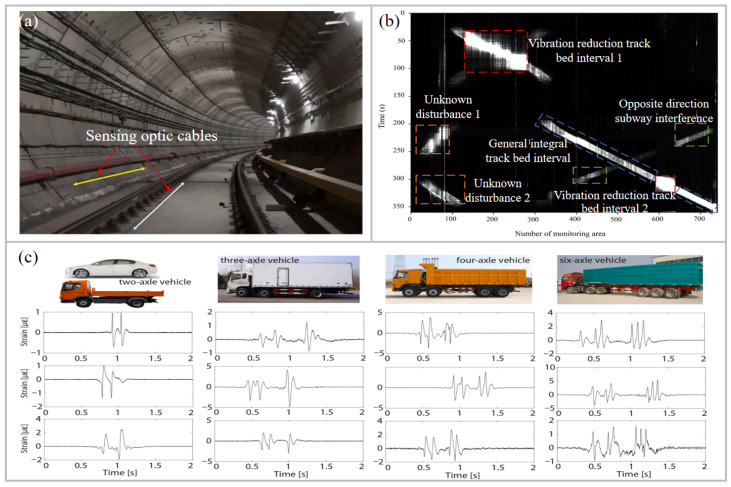
Application of UWFBG-based DAS in traffic and transportation. (**a**) Schematic diagram of the DAS layout of the track bed (white arrow) and tunnel wall (yellow arrow) [118]. (**b**) Representative signals detected by the DAS system [118]. (**c**) Characteristic waveforms generated by common vehicles, categorized by the number of wheel axles [120]. Reproduced with permission from Liu et al. [118]. Copyright 2023 Elsevier Ltd. Reprinted with permission from Jiang et al. [120]. Copyright 2025 Elsevier Ltd.

In subway applications, DAS also monitors illegal intrusion and evaluates shock absorption. A UWFBG array collected vibration signals to detect intrusions by drilling rigs [116,117]. The alarm system identifies engine vibrations based on narrowband characteristics, and statistical analysis determines intrusion locations. Subway vibration reduction was also assessed, as train-induced vibrations could significantly impact buildings and residents [118]. Figure 9a shows two parallel fiber-optic cables on tracks and tunnel walls to collect real-time train vibrations. Figure 9b shows a waterfall chart of vibration monitoring data obtained from a 3.7 km UWFBG array. The detected signals include those from vibration reduction track beds, passing trains, and other types of signals. The reduction effect was quantified using Spearman’s correlation coefficient.

Vehicle detection is essential for highway traffic monitoring, management, and intelligent services. A long-distance real-time highway vehicle detection method using UWFBG arrays buried optical cables 30 cm underground at lane centers. This approach achieves lane-level detection [119]. Vehicle trajectories were reconstructed using spatiotemporal data. The vibration characteristics of some common vehicles with different wheel axle numbers are shown in Figure 9c [120].

### 5.4. Structural Health Monitoring

**Figure 10:**
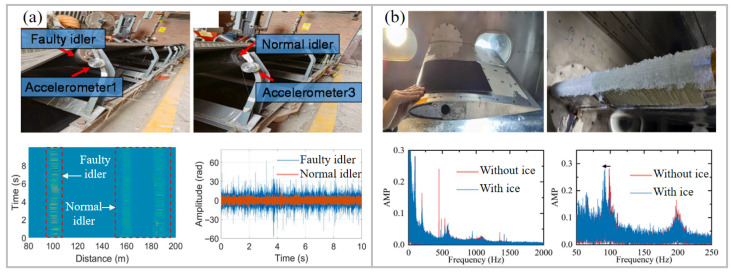
Application of UWFBG-based DAS in structural health monitoring. (**a**) Conveyor belt monitoring, showing the optical fiber sensor layout and a representative measured waveform [122]. (**b**) Aircraft wing monitoring, showing the wing model and the vibration spectra under both iced and ice-free conditions [123]. Reproduced with permission from Zheng et al. [122]. Copyright 2024 Elsevier Ltd. Reproduced with permission from Gui et al. [123]. Copyright 2025 Elsevier Ltd.

DAS is commonly used for structural health monitoring in engineering fields. In 2023, DAS was used to measure acoustic, dynamic, and static strain variations along a steel I-beam generated by an impact load [121]. By recovering the strain curve, the damping constant and natural frequency of the damping resonator can be measured, providing data for structural health monitoring. In mines, the safety of belt conveyors is crucial. Shutdown failures often stem from damaged idlers [122]. Considering the large number and dense distribution of idlers, the QDAS system based on a UWFBG array is suitable. Figure 10a shows the layout of the optical fiber and the measured vibration waveforms of faulty and normal idlers. The vibration signals collected by the QDAS system were classified using an automatic fault classification algorithm based on self-supervised learning (SSL). In the seven types of fault classification tasks, only 3.6% of labeled data was needed to achieve a diagnostic accuracy of 95.37%. In the airplane industry, ice on aircraft wings is a significant threat to flight safety [123]. The icy conditions of wings could be accurately identified with real-time tracking of the resonance frequency. An icing test conducted in an ice wind tunnel revealed a clear correlation between the wing vibration frequency and the icing state, as illustrated in Figure 10b. In addition, the system achieved high-precision temperature measurement to prevent excessive heating during de-icing by capturing the phase noise of the laser.

### 5.5. Hydrophone

**Figure 11:**
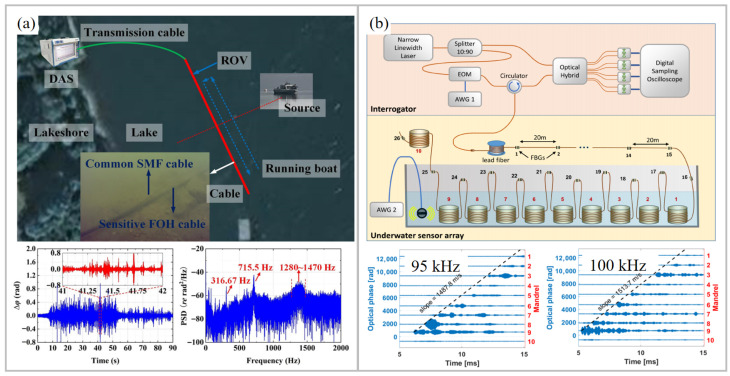
Application of UWFBG-based DAS in hydrophone. (**a**) Configuration of hydrophones and the corresponding sound profiles recorded from underwater remotely operated vehicles [126]. (**b**) Schematic of a hydrophone-integrated DAS system and the measured underwater acoustic waveforms at 95 kHz and 100 kHz [82]. FOH: fiber-optic hydrophone; ROV: remotely operated vehicles. Reprinted with permission from Chen et al. [126]. Copyright 2023 Elsevier Ltd. Reprinted with permission from Arbel et al. [82]. Copyright 2024 Institute of Electrical and Electronics Engineers Inc.

DAS technology has been widely applied in the field of hydrophones [124,125]. To improve the sensitivity and frequency flatness of the DAS hydrophone, acoustic-sensitive structures have been studied. An ultra-lightweight and high-sensitivity DAS hydrophone cable with an all-solid-state multi-layer composite structure was proposed [126]. The hydrophone had a sensitivity of −137.2 dB re rad/(μPa·m) and a flat frequency response from 5 Hz to 2 kHz. Its acoustic sensitivity at 1 Hz was −125.3 dB re rad/(μPa·m). The equivalent sound pressure level of the system at 1 kHz was lower than Deep Sea State Zero. In field testing, the system successfully detected and tracked invasive targets such as underwater remote operated vehicles and frogmen, as shown in Figure 11a. Moreover, elastic theory and finite element analysis were applied to optimize the structure of the mandrel [127]. The fiber winding ratio was optimized through mathematical simulation to achieve an omni-directional fiber-optic hydrophone. Experimental results showed that the designed hydrophone had a flatness of ±1.5 dB in the frequency range from 10 Hz to 1000 Hz, an average sensitivity of −113 dB re rad/µPa with a spatial resolution of 20 m, and a horizontal directivity of ±1 dB at 1 kHz. Its average minimum detectable sound pressure was as low as 14.1 µPa.

In 2022, a DAS system based on UWFBG arrays and a 3 × 3 coupler demodulation algorithm was used as a hydrophone. The intensity difference in the 3 × 3 coupler’s interference signals was compensated through cubic spline interpolation [128]. A hydrophone array containing 800 sensors was constructed using a UWFBG array with a reflectivity of −50 dB. The low-frequency underwater acoustic signals within 5–50 Hz generated by the vibrating liquid column method were recovered with high SNR. Moreover, the RIME-SVMD algorithm was used for the background noise in DAS hydrophone systems [99]. A UWFBG hydrophone array was fabricated as a test array, wound into a 6-fiber ring, and freely suspended in an anechoic pool. This algorithm effectively eliminated system and environmental noise while retaining complete underwater acoustic information. In addition, the directional detection of UWFBG hydrophone arrays can be achieved by using beamforming technology. Furthermore, the C-AMI method was used to achieve underwater ultrasonic detection. The method enabled a Q-DAS hydrophone with a maximum length of 4 km and spatiotemporal tracking of ultrasonic pulses with carrier frequencies up to 100 kHz, as shown in Figure 11b [82].

### 5.6. Perimeter Security and Intrusion Incidents

Intrusion detection and identification have always been challenging in the field of perimeter security. An intrusion monitoring scheme based on the Gaussian Mixture Model (GMM) and Hidden Markov Model (HMM) was proposed [129]. This model simultaneously analyzed the characteristics and temporal dependencies of vibration signals detected by DAS systems. It accurately identified three types of invasive behaviors and two types of non-invasive behaviors, with an average recognition rate of 98.2%. Moreover, the demodulated signal can be converted into a high-dimensional random matrix [130]. Then, the statistical characteristics of the matrix according to the Marcenko–Pastur (M-P) law and ring law were analyzed to efficiently confirm the presence of intrusion events. By comparing monitoring results during normal and crusher operations, intrusion events could be detected within 4.5 s with an accuracy exceeding 90%.

**Figure 12:**
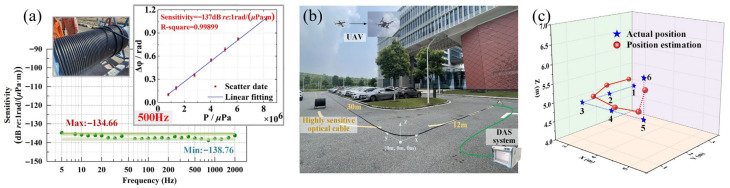
Application of UWFBG-based DAS in perimeter security and intrusion incidents. (**a**) Sensitivity of the sensing cables. (**b**) Configuration of the fiber-optic sensing array. (**c**) Drone flight path tracked by the DAS system [132]. Reproduced with permission from Fang et al. [132]. Copyright 2023 Institute of Electrical and Electronics Engineers Inc.

In recent years, the widespread use of drones has raised serious concerns about security and privacy protection. DAS has shown advantages in long-distance vibration monitoring, but its sensitivity to detecting weak airborne acoustic signals is insufficient. For this purpose, dispersed enhanced fiber-optic acoustic sensors (FOASs) were developed for DAS-based detection of drone sounds [131]. The FOAS sensor exhibited an ultra-high sensitivity of −101.21 dB re rad/µPa with a spatial resolution of 4 m. By connecting four FOASs in series to form a sensing array and combining it with a camera system, the enhanced view of the sound field and image fusion was successfully identified. In addition, continuous high-sensitivity sensing cables with a sensitivity of −137 dB re: 1 rad/(µPa·m) were fabricated using uniformly wrapped scattering-enhanced optical fibers, as shown in Figure 12a [132]. The collected drone sound signals were segmented into continuous frames and subjected to denoising. The Time Difference of Arrival (TDOA) algorithm based on Generalized Cross Correlation (GCC) and Least Squares Estimation (LSE) methods was used for localization and tracking. The field tests layout is shown in Figure 12b. The results show that the system enabled drone positioning and tracking in low-altitude airspace with a positioning error of less than 0.5 m, as shown in Figure 12c.

## 6. Conclusions and Prospects

The UWFBG array exhibits significant advantages in DAS systems, owing to its unique scattering properties. This paper analyzes the scattering characteristics and sensing principles of UWFBG arrays in DAS. A review of the fabrication methods for large-scale UWFBG arrays is provided, including the DTG technique, UV exposure using fibers with UV-transparent coatings, and the fs laser inscription method. Furthermore, the typical performance enhancements enabled by UWFBG arrays in DAS systems are discussed, primarily encompassing increased scattering intensity, fading suppression, improved frequency response, and PNC. Finally, the widespread engineering applications of scattering-enhanced optical fibers based on UWFBG and similar structures are highlighted, with examples in oil and gas exploration, pipeline integrity monitoring, intelligent transportation systems, structural health monitoring, hydroacoustics, and perimeter intrusion detection.

For future applications, non-contact monitoring scenarios, like drone intrusion detection, demand high sensitivity from DAS systems. This requirement underscores the importance of developing advanced packaging technologies for UWFBG optical cables in practical implementations. Additionally, the integration of artificial intelligence with this sensing technology is expected to expand its capabilities in intelligent pattern recognition for a broader range of fields.

## Figures and Tables

**Table 1:** Summary of UWFBG array fabrication technologies.

UWFBG Fabrication Technologies	Technical Characteristics	Advantages	Typical Applicable Scenarios
DTG technology	In situ UV inscription during fiber drawing before coating	Preserves fiber mechanical strength; avoids UV absorption by coating; and highly efficient fabrication	Distributed fiber sensing in large-scale and long-distance systems
UV exposure with UV-transparent coating fiber	UV phase mask inscription through UV-transparent coating	No need to strip coating; mature and stable fabrication technology	Distributed fiber sensing in conventional industrial environments
Fs laser direct writing technology	Fs laser inscription through standard coating	No reliance on fiber photosensitivity; high design flexibility; and high-temperature resistance	Distributed fiber sensing in extreme environments

## Data Availability

No new data were created or analyzed in this study. Data sharing is not applicable to this article.

## Abbreviations

The following abbreviations are used in this manuscript:

DAS	Distributed acoustic sensing
RBS	Rayleigh backscattering
SMF	Single-mode fibers
SNR	Signal-to-noise ratio
UWFBG	Ultra-weak fiber Bragg grating
UV	Ultraviolet
DOFS	Distributed optical fiber sensing
SEP	Scattering-enhanced point
FBG	Fiber Bragg grating
fs	Femtosecond
FUT	Fiber under test
COTDR	Coherent optical time-domain reflectometer
AOM	Acousto-optic modulator
LO	Local oscillator
PBS	Polarizing beam splitters
PGC	Phase generated carrier
QDAS	Quasi-distributed acoustic sensing
DTG	Drawing towers grating
PI	Polyimide
MCF	Multi-core fiber
MC	Microcavity
IFPI	Intrinsic Fabry-Perot interferometer
FSR	Free spectral range
CMCA	Composite microcavity arrays
PbP	Point-by-point
NA	Numerical aperture
Φ-OTDR	Phase-sensitivity optical time-domain reflectometer
USFBG	Ultra-short fiber Bragg grating arrays
CDPP	Composite double probe pulse ()
FDM	Frequency division multiplexing
WDM	Wavelength division multiplexing
TSM	Time-slot multiplexing
CDM	Code division multiplexing
OFDM	Orthogonal frequency division multiplexing
ICP	Interleaved chirped-pulses
IICP	Interleaved identical chirped pulses
C-AMI	Coded array matched interrogation
MFTSM	Multi-frequency time-slot multiplexing
EMD	Empirical mode decomposition
PNC	Phase noise compensation
LS-SVM	Least-squares support vector machine
OPD	Optical path difference
FMD	Feature modal decomposition
RIME-SVMD	RIME optimization algorithm and successive variational mode decomposition
IMF	Intrinsic mode function
VMD	Variational mode decomposition
VSP	Vertical seismic profiling
3C	Three-component
SD	Standard deviation
CEEMD	Complete ensemble empirical mode decomposition with adaptive noise
CNN	Convolutional neural network
SE	Squeeze-and-excitation
CAE	Convolutional autoencoder
SVD	Singular value decomposition
SSDA	Sequential similarity detection algorithm
GMM	Gaussian mixture model
HMM	Hidden Markov model
M-P	Marcenko-Pastur
FOAS	Fiber optic acoustic sensor
RMSE	Root mean square error ()
TDOA	Time difference of arrival
GCC	Generalized cross correlation
LSE	Least squares estimation
